# Relationship Between Metabolic Age Determined by Bioimpedance and Insulin Resistance Risk Scales in Spanish Workers

**DOI:** 10.3390/nu17060945

**Published:** 2025-03-08

**Authors:** Ignacio Ramírez-Gallegos, Pedro Juan Tárraga López, Hernán Paublini Oliveira, Ángel Arturo López-González, Cristina Martorell Sánchez, Emilio Martínez-Almoyna-Rifá, José Ignacio Ramírez-Manent

**Affiliations:** 1 ADEMA-Health Group University Institute of Health Sciences Research (IUNICS), 07120 Palma, Balearic Islands, Spain; ignacioramirezgallegos@gmail.com (I.R.-G.); h.paublini@eua.edu.es (H.P.O.); c.martorell@eua.edu.es (C.M.S.); emilio@mompra.com (E.M.-A.-R.); joseignacio.ramirez@ibsalut.es (J.I.R.-M.); 2 Faculty of Medicine, University of Castilla la Mancha, 02071 Albacete, Castilla-La Mancha, Spain; pjtarraga@sescam.jccm.es; 3 Faculty of Dentistry, University School ADEMA, 07009 Palma, Balearic Islands, Spain; 4 IDISBA, Balearic Islands Health Research Institute Foundation, 07010 Palma, Balearic Islands, Spain; 5 Balearic Islands Health Service, 07010 Palma, Balearic Islands, Spain; 6 Faculty of Medicine, University of the Balearic Islands, 07010 Palma, Balearic Islands, Spain

**Keywords:** metabolic age, insulin resistance, Mediterranean diet, physical activity, sociodemographic variables, smoking

## Abstract

**Introduction:** Metabolic age (MA) is the difference between an individual’s actual age and the age of their body based on physiological and biological factors. It is an indicator that reflects a person’s physical and biological state, regardless of chronological age. Insulin resistance (IR) is a health disorder in which tissues exhibit a reduced response to the circulating glucose uptake stimulated by insulin. **Objective:** The aim of this study is to evaluate the association between MA, determined through bioelectrical impedance analysis, and the risk of IR, assessed using validated scales, in a cohort of Spanish workers. **Methodology:** A descriptive cross-sectional study was conducted on 8590 Spanish workers to assess the association between MA and a set of sociodemographic variables, health habits, and IR risk scales such as the Triglyceride–Glucose Index (TyG Index), Metabolic Score for Insulin Resistance (METS-IR), and Single Point Insulin Sensitivity Estimator (SPISE). **Results:** All analyzed variables were associated with MA values, with the strongest associations observed for IR risk scale values (OR 4.88 [95% CI 4.12–5.65] for METS-IR, 4.42 [95% CI 3.70–5.15] for SPISE, and 3.42 [95% CI 2.97–3.87] for the TyG Index) and physical activity. **Conclusions:** Metabolic age is influenced by sociodemographic variables such as age, sex, and social class; health habits such as smoking, physical activity, and adherence to the Mediterranean diet; and by IR risk scale values.

## 1. Introduction

Chronological age, which refers to the number of years elapsed since birth, has traditionally been the primary measure for estimating an individual’s health and life expectancy. However, chronological age does not accurately reflect a person’s true health status, as some individuals may exhibit excellent health despite an advanced chronological age, while others, despite being younger, may suffer from various conditions associated with premature aging [1]. This leads to patients with advanced chronological age sometimes undergoing unnecessary treatment. In contrast, young people with multiple risk factors do not receive treatment due to their chronological age. This highlights the need to find a corrective parameter for age. Metabolic age (MA), which describes the body’s functional status in relation to its metabolism, has emerged as a crucial indicator for assessing overall health and well-being, particularly in relation to metabolic health, which is directly associated with chronic diseases and comorbidities. This concept provides a more detailed perspective on individual health, allowing for more personalized and effective interventions. MA is a concept used to assess the body’s metabolic efficiency in comparison to a population average across different ages [2]. Through various measurements, such as body composition [3], basal metabolic rate (BMR) [4], and other physiological parameters [5], an individual’s MA can be estimated.

BMR is the minimum energy required at rest to maintain vital functions and decreases by approximately 1–2% every 10 years in adults. This decline occurs mainly due to the reduction in muscle tissue, which is replaced by fat tissue. BMR is typically estimated using chronological age, height, weight, and sex. However, weight alone does not distinguish between fat mass and fat-free mass.

To improve BMR estimation, multiple studies have demonstrated the usefulness of bioelectrical impedance analysis (BIA), which differentiates between fat-free mass and skeletal muscle mass [6,7,8,9]. These measurements determine that fat mass only influences a modification of between 2 and 3% of the BMR [10]. For its part, the company TANITA demonstrated that BMR calculated by bioimpedance was more precise than if determined by body weight or BMI [11]. In recent years, different authors have found that BIA provides better information on body composition than anthropometry, and good agreement with dual X-ray absorptiometry for determining fat mass [12,13,14]. More recently, Cretescu et al. (2025) found that BIA has a strong correlation with body fat percentage [15].

MA was developed as a method to explain the relationship between an individual’s BMR compared to that of others of the same chronological age. Elguezabal-Rodelo et al. (2021) demonstrated that MA is a better risk marker than most patients can easily perceive. Thus, if a person’s MA is higher than their chronological age, it indicates that their BMR is diminished, reflecting a higher cardiovascular risk [16].

This indicator provides a more comprehensive perspective on metabolic health status than simply considering chronological age [17,18]. According to some authors, a higher MA is associated with increased risks of metabolic diseases [17], reduced quality of life [19], and premature mortality [20].

The determination of MA is generally performed using bioimpedance devices that assess body composition [21]. These devices calculate BMR, which represents the amount of energy the body requires at rest to maintain vital functions [22]. This rate is then compared with demographic data to estimate MA. The technology employed in these devices has advanced significantly, enabling more accurate and accessible measurements for the general population [23].

An MA greater than chronological age may indicate an unhealthy lifestyle, including factors such as poor diet [24], lack of physical activity [25], or chronic stress [26]. These factors contribute to the deterioration of metabolic health and may lead to conditions such as obesity [27], type 2 diabetes (DM2) [28,29], and cardiovascular diseases [16,30]. Regular evaluation of MA could serve as a preventative tool [29] and a motivator for adopting healthier habits [4].

Insulin resistance (IR) is a condition in which the body’s cells do not respond adequately to insulin, a hormone crucial for glucose metabolism. This resistance forces the pancreas to produce more insulin to maintain normal blood glucose levels [31]. Over time, IR can lead to a range of metabolic disorders, including metabolic syndrome [32] and type 2 diabetes [33].

The development of IR is influenced by a combination of genetic [34] and socio-environmental factors [35,36]. Obesity, particularly visceral fat accumulation, is one of the primary risk factors [37,38]. Chronic inflammation [39] and oxidative stress [40] associated with excess adipose tissue contribute to impaired insulin signaling in cells. Additionally, a diet high in sugars and saturated fats [41] and a sedentary lifestyle [42] are critical factors in the development of this condition.

The relationship between MA and IR is close and bidirectional. A higher MA is often associated with a greater prevalence of IR. This is because many of the factors that increase MA, such as obesity and physical inactivity, are also significant contributors to IR. Studies have shown that individuals with an MA exceeding their chronological age exhibit elevated levels of inflammatory markers and disruptions in glucose homeostasis, indicating underlying IR [43].

The high prevalence of IR and elevated MA in the general population has a significant impact on public health [44]. These conditions not only increase the risk of metabolic diseases but also lead to higher healthcare costs and reduced quality of life for affected individuals [45]. Public health policies promoting nutritional education, physical exercise, and stress management are crucial to addressing these issues [46].

To reduce MA and improve insulin sensitivity, it is essential to implement intervention strategies targeting modifiable factors. Regular exercise programs, particularly those combining aerobic and resistance training, have been shown to effectively improve body composition and metabolic function [47]. Furthermore, a balanced diet rich in fiber, lean proteins, healthy fats, and low in refined sugars is fundamental for improving metabolic health [48].

Stress management and sleep quality are also critical components in managing MA and IR. Chronic stress [49] and sleep deprivation [50] are associated with metabolic dysfunction and increased IR due to hormonal and autonomic nervous system disturbances. Stress management techniques such as meditation [51], yoga [52], and cognitive-behavioral therapy [53], alongside promoting healthy sleep habits [54], can significantly contribute to improving metabolic health.

Research in the field of MA and IR is continuously evolving. New technologies and analytical approaches, such as genomics and metabolomics, are providing deeper insights into the molecular and genetic bases of these conditions [55]. Additionally, studies on the gut microbiome are revealing its crucial role in regulating metabolism and IR, opening new avenues for therapeutic interventions [56].

The objective of this study is to assess the association between MA, determined through bioimpedance, and the risk of IR, assessed using validated scales, in a cohort of Spanish workers.

## 2. Materials and Methods

### 2.1. Participants

This study employed a cross-sectional, descriptive design, encompassing a cohort of 8590 Spanish workers from the Balearic Islands. The participants were individuals who underwent their annual occupational health check-up between January 2019 and December 2020, facilitated by an occupational health and risk prevention service. This service is utilized by various companies across sectors such as healthcare, public administration, hospitality, retail, transportation, education, industry, and cleaning.

**Figure 1:**
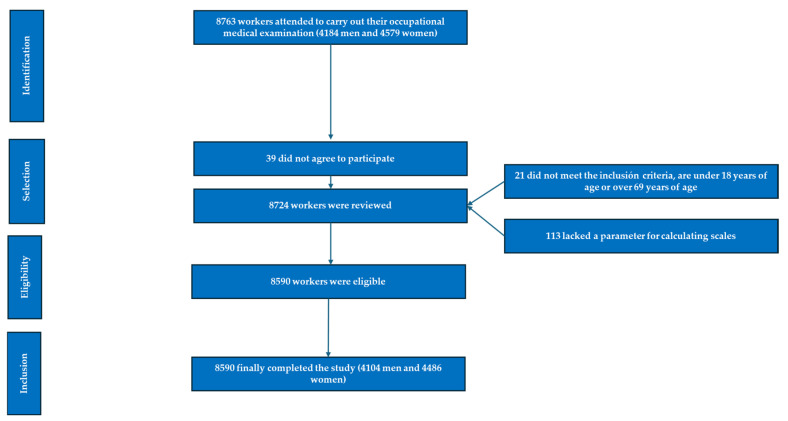
PRISMA flowchart of participants in this study.

Additional details are provided in the flowchart in Figure 1.

Inclusion Criteria:

- Individuals aged 18–69 years.
- Voluntary participation in the study.
- Consent to the use of personal data for epidemiological purposes.
- Employment within one of the participating companies, without being on temporary disability leave during the study period.

Exclusion Criteria:

- Individuals below 18 or above 69 years of age.
- Non-employees of participating companies.
- Refusal to participate in the study.
- Declined consent for data usage in epidemiological studies.
- Missing parameters required for scale calculations.
- Patients diagnosed with diabetes mellitus.

### 2.2. Variable Determination

Data collection for this study was undertaken by healthcare personnel affiliated with the occupational health services of the participating companies. The following methodologies were used:

Anamnesis:

A comprehensive clinical history was obtained, including sociodemographic details such as age, gender, social class, smoking habits, physical activity levels, and adherence to the Mediterranean diet.

Anthropometric and Clinical Measurements:

Measurements included height, weight, waist circumference, hip circumference, and systolic and diastolic blood pressure.

Laboratory Analyses:

Blood samples were analyzed for lipid profiles and blood glucose levels.

#### 2.2.1. Anthropometric Measurements

To reduce potential biases, all measurement protocols were standardized. Height and weight were recorded using a SECA 700 scale (SECA, Chino, CA, USA) and SECA 220 stadiometer (SECA, Chino, CA, USA), with participants in light clothing and barefoot, following ISAK guidelines for anthropometric assessments [57]. Measurements were documented in centimeters and kilograms.

Waist circumference was measured with a SECA tape (SECA, Chino, CA, USA), placed midway between the last rib and the iliac crest, parallel to the floor, with participants standing relaxed. Hip circumference was measured similarly, using the widest point of the hips.

#### 2.2.2. Clinical Measurements

Blood pressure was assessed using an OMRON-M3 monitor (OMRON, Osaka, Japan). Participants were seated with their back supported, arm resting at heart level, and legs uncrossed, following a minimum rest period of 10 min. To ensure accuracy, participants were instructed to abstain from food, alcohol, caffeine, smoking, or tea consumption for at least one hour before measurement. The cuff was fitted 2–3 cm above the elbow crease, with various sizes available for individual needs. Three consecutive readings were taken at one-minute intervals, and the final blood pressure value was determined as the average of the three.

#### 2.2.3. Laboratory Analyses

Blood samples were collected via venipuncture after a 12 h fasting period. The samples were refrigerated and processed within 48–72 h to maintain integrity. Lipid profiles (triglycerides, total cholesterol, HDL cholesterol) and blood glucose levels were determined using enzymatic methods. LDL cholesterol was calculated using the Friedewald formula [58] unless triglycerides exceeded 400 mg/dL, in which case direct measurement was performed. All laboratory results are expressed in mg/dL.

#### 2.2.4. Risk Scales

Adherence to the Mediterranean Diet:

The 14-item PREDIMED questionnaire was utilized, scoring each item as 0 or 1. Scores ≥ 9 indicated strong adherence to the Mediterranean diet [59].

Physical Activity Assessment:

Physical activity levels were evaluated using the International Physical Activity Questionnaire (IPAQ), which assesses activity during the previous seven days [60].

Smoking Status:

Participants were classified as smokers if they had consumed at least one cigarette daily (or its equivalent) in the past 30 days or had quit smoking within the preceding 12 months. Non-smokers included individuals who had abstained for more than a year or had never smoked.

Socioeconomic Classification:

Socioeconomic status was determined following the Spanish Society of Epidemiology’s 2011 guidelines. Class I included managers, directors, and university-educated professionals; Class II encompassed intermediate professionals and self-employed workers; and Class III consisted of manual laborers [61].

Metabolic Age and Avoidable Lost Life Years (ALLYs):

Metabolic age was assessed using a TANITA MC-780 S MA bioimpedancemeter (TANITA Corporation, Tokyo, Japan). ALLYs were calculated by subtracting chronological age from metabolic age. A difference of ≥12 years is associated with reduced cardiovascular risk. ALLYs were categorized as low (<3 years), normal (3–11 years), or high (≥12 years) [62].

Bioimpedance measurement, like all other measurements and assessments, was performed on the same day as the blood samples were taken. Therefore, all subjects were fasting for 12 h.

The insulin resistance risk scales that have been calculated, as well as their cut-off points, are presented in Table 1.

### 2.3. Statistical Analysis

Categorical variables were analyzed descriptively by computing their frequencies and distributions. For quantitative variables following a normal distribution, the mean and standard deviation were reported. Mean comparisons were performed using Student’s *t*-test, while proportion differences were assessed using the chi-square test.

In the multinomial logistic regression analysis, the dependent variable was defined as elevated values across the three insulin resistance risk scales. Independent variables were selected based on their biological relevance and statistical significance as identified in the existing literature. A multinomial logistic regression model was applied, with odds ratios calculated and model fit assessed using the Hosmer–Lemeshow test.

Stratified analyses conducted to control for potential confounding factors did not reveal significant effects. All statistical analyses were performed using SPSS software version 29.0, with a significance threshold set at 0.05.

## 3. Results

The anthropometric and clinical characteristics of the participants are summarized in Table 2. A total of 8590 individuals participated in the study, comprising 4104 men (47.8%) and 4486 women (52.2%). The mean age of the sample was slightly above 41 years, with the majority of participants falling within the 30–49 age range.

A comparative analysis of anthropometric, clinical, and biochemical variables revealed that females consistently exhibited lower values across all assessed parameters. The majority of the participants were classified within social class I.

Regarding smoking habits, approximately 15% of participants in both sexes were identified as current smokers. Physical inactivity was observed in 25.9% of men and 35.1% of women, indicating a higher prevalence of sedentary behavior among females. Notably, more than half of the participants of both sexes reported adherence to the Mediterranean diet.

Student’s *t*-test for means and chi square test were used for prevalence.

Table 3 and Table 4 present the mean values and the prevalence of elevated metabolic age (MA) based on various sociodemographic variables (age and social class), health habits (smoking, adherence to the Mediterranean diet, and physical activity), and insulin resistance (IR) risk scales. Both the mean values and the prevalence of elevated MA increase progressively with advancing age, decreasing socioeconomic status, and poor health habits (smokers, sedentary individuals, and those with low adherence to the Mediterranean diet). Higher MA values were also observed in individuals with elevated scores on the IR risk scales. In all cases, the mean values and prevalence of elevated MA were significantly higher in men. All observed differences were statistically significant (*p* < 0.001).

Table 5 displays the results of the multinomial logistic regression analysis. All the independent variables analyzed were associated with elevated MA values. Among these, the variables most strongly associated with higher odds ratios (ORs) were the IR scales, physical activity, and adherence to the Mediterranean diet. In every case, the observed differences were statistically significant (*p* < 0.001).

**Figure 2:**
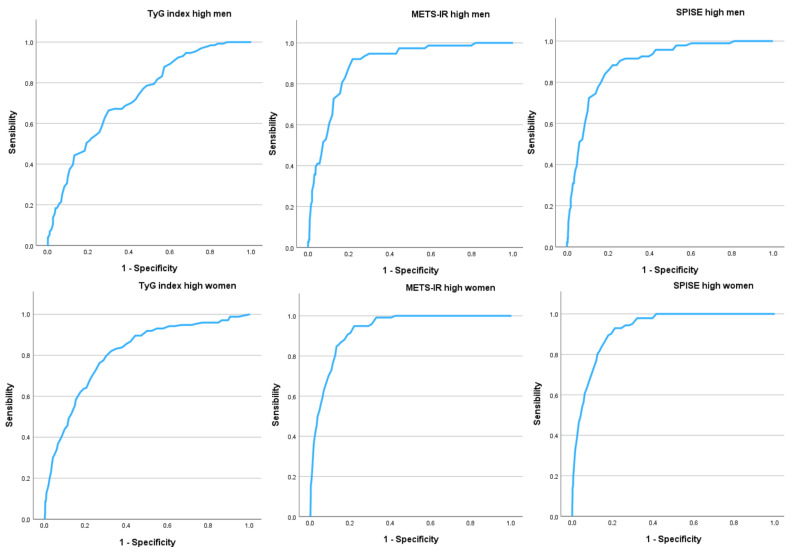
ROC curves. TyG: Triglyceride–Glucose Index. METS-IR: Metabolic Score for Insulin Resistance. SPISE-IR: Single Point Insulin Sensitivity Estimator.

Figure 2 and Table 6 illustrate the results of the ROC curves. The areas under the curve (AUC) were notably high, particularly for METS-IR and SPISE, with values close to or exceeding 0.900. In all cases, the AUC values were higher among women.

## 4. Discussion

This study provides robust evidence on how various sociodemographic, behavioral, and health-related variables are significantly associated with metabolic age (MA), an emerging clinical marker that estimates biological aging relative to chronological aging. The findings highlight that age, sex, socioeconomic status, smoking, adherence to the Mediterranean diet (MD), physical activity, and insulin resistance (IR) risk scales are key determinants in the variation in MA values.

As expected and consistent with existing literature, chronological age demonstrated a direct and significant association with MA values, serving as a strong predictor in the multivariable model. As age increases, so does the likelihood of elevated MA values, reflecting the cumulative impact of physiological and metabolic processes underlying aging [64]. Previous studies have documented that age-related functional and metabolic decline, such as reduced insulin sensitivity [63,65], increased visceral adiposity [66,67,68], and decreased lean mass [69], contributes to this relationship. However, the observed interindividual variability in MA within each age group underscores that metabolic aging is not a uniform process but is modulated by additional factors, including lifestyle and social conditions [24].

In this study, men exhibited significantly higher MA values and a greater prevalence of elevated values compared to women, suggesting a less favorable metabolic profile in males. This difference may be partly explained by hormonal factors, as premenopausal women benefit from estrogen’s protective effects on metabolism [70], including improved insulin sensitivity [71], a more favorable lipid profile [72], and lower visceral fat accumulation [73]. On the other hand, differences in health behaviors may also play a role, as men tend to have higher smoking rates [74] and lower adherence to healthy dietary patterns such as the MD [75], both of which contribute to accelerated metabolic aging.

Socioeconomic status (SES), measured in this study through social class classification, was inversely associated with MA values, with individuals in lower social classes exhibiting significantly higher average MA values and a greater prevalence of elevated values. This finding reinforces the notion that social inequalities profoundly affect metabolic health [76], likely through mechanisms including unequal access to healthcare resources, differential exposure to risk factors such as chronic stress and food insecurity, and barriers to adopting healthy behaviors [77]. Furthermore, individuals in lower social classes tend to exhibit higher rates of obesity [78], physical inactivity [79], and consumption of low-quality diets [80], which may mediate the relationship between SES and MA.

Smoking, recognized as one of the primary modifiable risk factors for a variety of chronic diseases, was significantly associated with higher MA values in this study. Smokers not only exhibited a higher average MA but also a greater prevalence of elevated values compared to non-smokers. This effect can be attributed to the negative impact of smoking on cardiovascular function [81], oxidative stress [82], systemic inflammation [83], and insulin sensitivity [84], all of which contribute to accelerated metabolic aging. These results underscore the importance of implementing effective public health policies to reduce tobacco consumption as a key strategy to mitigate metabolic aging and its associated consequences.

Adherence to the MD, assessed through the PREDIMED questionnaire, showed a significant inverse association with MA values, highlighting the protective role of this dietary pattern against metabolic aging. This effect can be explained by the anti-inflammatory [85] and antioxidant properties of the diet [86], which includes a high intake of fruits, vegetables, legumes, whole grains, fish, and olive oil, as well as a low intake of red meats and ultra-processed foods. Previous studies have demonstrated that the MD improves insulin sensitivity [87], reduces visceral fat accumulation [88], and promotes a favorable lipid profile [89], factors that lower the risk of metabolic [90] and cardiovascular diseases [91]. Plant-based diets and the Mediterranean diet provide multiple health benefits due to their high content of fiber, complex carbohydrates, vitamins, minerals, and phytochemicals. Isoflavones, such as genistein, act on estrogen receptors and are commonly used in postmenopausal women. Genistein, the primary isoflavone in soy, has been shown to improve bone health, enhance endothelial function, and reduce vasomotor symptoms without adverse effects. A one-year treatment with 54 mg/day of pure genistein improved cardiac function and reduced the risk of diabetes and cardiovascular disease in postmenopausal women with metabolic syndrome [92]. Our findings support the recommendation to promote the MD as an effective intervention to enhance metabolic health and reduce disparities in metabolic aging.

In this section, it is important to refer to the known intestinal microbiota. The gut microbiota plays a key role in regulating metabolism and inflammation. Factors such as nutrient composition, meal frequency, and timing influence its balance, affecting baseline inflammatory tone. This state can be exacerbated by overnutrition (metainflammation) and aging (chronic inflammation), reflecting immune system activation in response to danger signals. Metainflammation, as a specific form of inflammation induced by excess nutrients, is associated with accelerated aging, which may influence MA. From a geroscience perspective, integrating these processes enables the development of combined biomarkers, facilitating preventive strategies and personalized medicine in older adults [93,94].

The relationships between adiponectin, leptin, visfatin, and traditional indicators of metabolic diseases suggest that these adipokines could serve as additional markers of IR. In recent years, adipose tissue has been identified as an active endocrine organ that secretes multiple adipokines with key functions in metabolic homeostasis.

Adiponectin, considered a protective adipokine, has been inversely associated with insulin resistance and cardiovascular risk. Its levels are typically reduced in obesity and metabolic syndrome, suggesting that its reduction could serve as an early indicator of metabolic dysfunction. In contrast, leptin, whose main function is to regulate appetite and energy balance, is elevated in individuals with obesity, reflecting a state of leptin resistance. This phenomenon has been linked to an increased cardiometabolic risk and poorer glucose control, reinforcing the idea that dysregulation of this hormone could contribute to the progression of insulin resistance.

On the other hand, visfatin, an adipokine predominantly secreted by visceral adipose tissue, has been associated with pro-inflammatory effects and increased metabolic dysfunction. Elevated levels in individuals with obesity, type 2 diabetes, and metabolic syndrome may contribute to the chronic low-grade inflammation characteristic of these pathological states. Although evidence regarding its exact role remains controversial, its association with traditional indicators of metabolic diseases suggests it could play a relevant role in the pathogenesis of insulin resistance. Together, these adipokines provide a broader framework for understanding the underlying mechanisms of insulin resistance and could complement traditional diagnostic tools, such as the HOMA-IR index, fasting glucose and insulin measurements, or insulin resistance risk scales [95].

Regular physical activity was identified as another determinant of MA values. Participants engaging in regular exercise exhibited significantly lower MA values and a lower prevalence of elevated values compared to sedentary individuals. Physical activity enhances insulin sensitivity [96], increases energy expenditure [97], reduces visceral adiposity [98], and helps preserve muscle mass [99], all of which are essential for maintaining a healthy metabolism and preventing accelerated metabolic aging [100]. Our results emphasize the importance of promoting accessible and sustainable physical activity programs for the entire population as a key strategy to improve metabolic health and reduce inequalities in MA.

Although our study excluded patients diagnosed with DM, we do not have a record of the drugs that the participants are taking for other pathologies. The effect that certain medications have on CV risk may have misled the association between MS. This is a limitation of the study that we note in Section 5.

Among all variables analyzed, IR risk scales (such as METS-IR and SPISE) showed the strongest association with elevated MA values, underscoring the critical role of insulin resistance as a key determinant of metabolic aging. Individuals with high scores on these scales exhibited not only a significantly higher average MA but also a greater prevalence of elevated values. IR is closely linked to metabolic syndrome and other chronic conditions such as type 2 diabetes [101], cardiovascular disease [102], and obesity [39], which accelerate biological aging through inflammatory and oxidative mechanisms [103]. Our findings highlight the need to incorporate these scales as useful tools for identifying at-risk individuals and prioritizing interventions aimed at improving insulin sensitivity, such as dietary and physical activity modifications.

A particularly relevant finding of this study was the interaction between the analyzed variables and sex. Although women generally exhibited lower MA values, the observed differences in factors associated with MA, such as physical activity and the MD, suggest that the benefits of these interventions may vary by sex. For instance, women’s higher adherence to the MD and lower smoking prevalence may contribute to their more favorable metabolic profile, whereas men may benefit more from targeted interventions to improve specific habits, such as smoking cessation and increased physical activity. These findings underscore the importance of adopting personalized, gender-sensitive approaches in the prevention and treatment of metabolic aging.

The findings of this study have significant clinical and public health implications. First, the use of metabolic age (MA) as a marker for assessing metabolic aging enables the identification of individuals at high risk of developing metabolic and cardiovascular diseases before clinical manifestation. Insulin resistance (IR) is an increasing public health concern due to its high prevalence and its impact on the development of chronic diseases. The association with an elevated MA may serve as an early detection tool, helping to identify at-risk individuals before they develop severe complications. This is particularly crucial in a context where type 2 diabetes and cardiovascular diseases pose a significant burden on healthcare systems, both in terms of costs and quality of life.

Second, our observations highlight the need to address the social and behavioral determinants of metabolic health, including the promotion of healthy lifestyles and the reduction in social inequalities. The use of MA in clinical practice and public health could enhance prevention strategies by providing a more individualized approach. For instance, in populations with a high prevalence of obesity and sedentary behavior, monitoring MA alongside insulin resistance indicators could facilitate the implementation of early intervention programs. These programs could focus on lifestyle modifications, such as improving dietary habits and increasing physical activity, before metabolic diseases emerge.

Third, the strong association between IR scales and MA suggests that these tools should be integrated into clinical practice as part of a comprehensive approach to metabolic risk assessment. If an elevated MA is detected in a patient with signs of insulin resistance, early intervention strategies can be implemented, thereby reducing the progression to more severe diseases. Its use in public health could improve the identification of at-risk populations and optimize prevention and treatment strategies, ultimately reducing the burden of chronic diseases and enhancing individuals’ quality of life.

## 5. Strengths and Limitations

One of the strengths of this study is the large sample size, comprising nearly 8600 individuals, and the wide variety of variables analyzed.

While this study provides a comprehensive overview of the determinants of MA, several limitations should be considered:

Only individuals of working age (18–69 years) were included, excluding unemployed individuals, retirees, minors, and those over 69 years. Therefore, our results cannot be generalized to the entire population, as certain age groups are missing.

Over half of the sample belongs to social class I, which may not be representative of the general population.

As the study focused exclusively on the population of the Balearic Islands, the findings may differ for other populations and thus cannot be extrapolated.

Although a wide range of variables was analyzed, other potentially relevant factors, such as comorbidities, pharmacological treatments, chronic stress, and sleep quality, were not evaluated due to data unavailability. Future research should explore these areas and conduct longitudinal studies to confirm the directionality of the observed associations.

Another limitation is the “healthy worker effect”, a common methodological issue in occupational health studies. Workers with chronic illnesses or greater predisposition to disease may be less likely to attend occupational health check-ups, potentially underestimating the results.

Being a cross-sectional study, it establishes associations between the variables and obesity but does not establish causality.

Finally, the menstrual phase of women included in the study was not accounted for, which could lead to weight variations.

## 6. Conclusions

MA and IR are critical indicators of overall metabolic health. Understanding the interrelation of these factors and the underlying mechanisms influencing them is essential for developing effective prevention and treatment strategies. Interventions addressing diet, exercise, stress management, and sleep, alongside a focus on continued research, are crucial for improving metabolic health and reducing the burden of associated diseases in the population.

## Figures and Tables

**Table 1:** Formulas and cut-off of different insulin resistance risk scales.

	Formula	Cut-Off
TyG Index [63]	LN (triglycerides × glycaemia/2)	>8.5
METS-IR [63]	LN (2× Glycaemia + triglycerides) × BMI/LN(HDL-c)	>50
SPISE [63]	(=600 × HDL ^0.185^/Triglycerides ^0.2^ × BMI ^1.338^)	6.14

TyG: Triglyceride–Glucose Index. METS-IR: Metabolic Score for Insulin Resistance. SPISE: Single Point Insulin Sensitivity Estimator.

**Table 2:** Characteristics of participants.

	Men *n* = 4104	Women *n* = 4486	
	Mean (SD)	Mean (SD)	*p*-Value
Age (years)	41.6 (10.6)	41.5 (10.5)	0.492
Height (cm)	175.8 (7.2)	162.5 (6.1)	<0.001
Weight (kg)	81.2 (14.8)	63.9 (13.6)	<0.001
Waist circumference (cm)	89.8 (12.5)	77.0 (12.0)	<0.001
Hip circumference (cm)	101.8 (8.7)	99.6 (10.9)	<0.001
Systolic blood pressure (mmHg)	128.6 (13.3)	117.2 (14.1)	<0.001
Diastolic blood pressure (mmHg)	79.9 (10.2)	74.9 (9.9)	<0.001
Glycaemia (mg/dL)	93.4 (17.8)	88.9 (12.6)	<0.001
Total cholesterol (mg/dL)	191.8 (36.0)	189.0 (34.8)	<0.001
HDL-c (mg/dL)	49.2 (11.3)	59.5 (12.8)	<0.001
LDL-c (mg/dL)	124.0 (54.6)	113.8 (30.7)	<0.001
Triglycerides (mg/dL)	107.8 (69.4)	81.5 (46.3)	<0.001
GGT (UI)	31.5 (30.0)	18.5 (15.9)	<0.001
AST (UI)	24.4 (17.3)	18.2 (7.7)	<0.001
ALT (UI)	29.3 (34.9)	17.3 (13.4)	<0.001
	**%**	**%**	***p*-value**
18–29 years	15.5	16.8	0.005
30–39 years	27.8	25.1	
40–49 years	32.7	34.4	
50–59 years	19.0	19.7	
60–69 years	5.0	4.0	
Social class I	57.1	50.8	<0.001
Social class II	20.2	23.8	
Social class III	22.7	25.4	
Non-smokers	84.5	84.2	0.348
Smokers	15.5	15.8	
Non-physical activity	25.9	35.1	<0.001
Physical activity 1–3 days/week	27.0	26.5	
Physical activity more 3 days/week	47.1	38.4	
Non-Mediterranean diet	44.5	41.6	<0.001
Mediterranean diet	55.5	58.4	

SD: standard deviation. HDL-c: high-density lipoprotein. LDL-c: low-density lipoprotein. GGT: gamma-glutamyl transferase. AST: aspartate aminotransferase. ALT: alanine aminotransferase.

**Table 3:** Mean values of metabolic age according to values of sociodemographic variables, healthy habits and insulin resistance risk scales by sex.

		Men			Women	
Metabolic Age	*n*	Mean (SD)	*p*-Value	*n*	Mean (SD)	*p*-Value
18–29 years	636	−4.7 (10.1)	<0.001	754	−6.0 (10.7)	<0.001
30–39 years	1140	−4.3 (11.0)		1126	−5.2 (9.8)	
40–49 years	1344	−4.1 (11.0)		1544	−4.8 (11.4)	
50–59 years	780	−2.3 (11.3)		882	−4.7 (11.5)	
60–69 years	204	−1.5 (11.4)		180	−4.3 (10.2)	
Social class I	2346	−5.5 (10.4)	<0.001	2278	−7.5 (9.6)	<0.001
Social class II	828	−2.3 (10.6)		1068	−3.0 (11.9)	
Social class III	930	−0.8 (12.0)		1140	−2.7 (11.8)	
Non-smokers	3468	−4.1 (10.9)	<0.001	3776	−5.3 (10.9)	<0.001
Smokers	636	−1.7 (11.3)		710	−4.8 (11.4)	
Non-physical activity	1062	3.1 (10.9)	<0.001	1574	−0.7 (11.9)	<0.001
Physical activity 1–3 days/week	1110	−3.4 (10.1)		1187	−5.9 (10.0)	
Physical activity more 3 days/week	1932	−7.8 (9.5)		1725	−8.7 (9.2)	
Non-Mediterranean diet	1827	0.1 (11.9)		1866	−3.3 (11.8)	
Mediterranean diet	2277	−6.8 (9.1)		2620	−6.5 (10.2)	
TyG Index normal	3318	−5.2 (10.5)	<0.001	4140	−6.0 (10.6)	<0.001
TyG Index high	786	2.1 (11.2)		346	4.1 (11.1)	
METS-IR normal	3650	−5.7 (9.8)	<0.001	4250	−6.2 (10.3)	<0.001
METS-IR high	454	11.4 (7.7)		236	13.7 (4.2)	
SPISE normal	3540	−6.1 (9.5)	<0.001	4202	−6.4 (10.2)	<0.001
SPISE high	564	10.8 (8.1)		284	13.3 (4.8)	

TyG: Triglyceride–Glucose Index. METS-IR: Metabolic Score for Insulin Resistance. SPISE: Single Point Insulin Sensitivity Estimator. SD: standard deviation.

**Table 4:** Prevalence of high values of metabolic age according to values of sociodemographic variables, healthy habits and insulin resistance risk scales by sex.

		MA High Men			MA High Women	
Metabolic Age	*n*	%	*p*-Value	*n*	%	*p*-Value
18–29 years	636	18.9	<0.001	754	19.6	<0.001
30–39 years	1140	24.7		1126	19.7	
40–49 years	1344	27.7		1544	23.3	
50–59 years	780	28.5		882	23.6	
60–69 years	204	31.0		180	25.4	
Social class I	2346	19.9	<0.001	2278	14.7	<0.001
Social class II	828	26.1		1068	30.3	
Social class III	930	39.4		1140	32.6	
Non-smokers	3468	24.7	<0.001	3776	22.6	<0.001
Smokers	636	30.2		710	25.1	
Non-physical activity	1062	49.2	<0.001	1574	38.4	<0.001
Physical activity 1–3 days/week	1110	23.2		1187	18.0	
Physical activity more 3 days/week	1932	14.0		1725	12.4	
Non-Mediterranean diet	1827	40.4		1866	29.7	
Mediterranean diet	2277	13.7		2620	18.2	
TyG Index normal	3318	21.0	<0.001	4140	20.4	<0.001
TyG Index high	786	45.0		346	53.8	
METS-IR normal	3650	17.8	<0.001	4250	19.0	<0.001
METS-IR high	454	88.1		236	95.8	
SPISE normal	3540	15.9	<0.001	4202	18.1	<0.001
SPISE high	564	86.2		284	95.1	

MA: metabolic age. TyG: Triglyceride–Glucose Index. METS-IR: Metabolic Score for Insulin Resistance. SPISE: Single Point Insulin Sensitivity Estimator.

**Table 5:** Multinomial logistic regression.

	MA High	MA High	MA High
	OR (95% CI)	OR (95% CI)	OR (95% CI)
Women	1	1	1
Men	1.13 (1.10–1.17)	1.09 (1.06–1.12)	1.15 (1.10–1.21)
18–29 years	1	1	1
30–39 years	1.15 (1.12–1.18)	1.20 (1.16–1.24)	1.24 (1.19–1.30)
40–49 years	1.29 (1.24–1.35)	1.31 (1.26–1.37)	1.45 (1.38–1.52)
50–59 years	1.42 (1.34–1.50)	1.58 (1.49–1.68)	1.49 (1.40–1.59)
60–69 years	1.45 (1.37–1.53)	1.73 (1.62–1.84)	1.55 (1.45–1.66)
Social class I	1	1	1
Social class II	1.42 (1.33–1.51)	1.11 (0.07–1.16)	1.18 (1.12–1.25)
Social class III	2.58 (2.27–2.89)	2.45 (2.12–4.79)	2.38 (2.06–2.71)
Non-smokers	1	1	1
Smokers	1.07 (1.05–1.10)	1.08 (1.05–1.12)	1.07 (1.05–1.10)
Physical activity more 3 days/week	1	1	1
Physical activity 1–3 days/week	1.21 (1.17–1.26)	1.75 (1.60–1.91)	1.38 (1.30–1.47)
Non-physical activity	3.66 (3.21–4.12)	3.16 (2.90–3.43)	3.10 (2.61–3.60)
Mediterranean diet	1	1	1
Non-Mediterranean diet	2.12 (1.89–2.35)	2.33 (2.02–2.64)	2.22 (1.98–2.47)
TyG Index normal	1		
TyG Index high	3.42 (2.97–3.87)		
METS-IR normal		1	
METS-IR high		4.88 (4.12–5.65)	
SPISE normal			1
SPISE high			4.42 (3.70–5.15)

MA: metabolic age. TyG: Triglyceride–Glucose Index. METS-IR: Metabolic Score for Insulin Resistance. SPISE: Single Point Insulin Sensitivity Estimator. OR: odds ratio.

**Table 6:** ROC curves.

	Men *n* = 4104	Women *n* = 4486
	AUC (95% CI)	AUC (95% CI)
TyG Index high	0.679 (0.658–0.701)	0.742 (0.715–0.769)
METS-IR high	0.888 (0.870–0.906)	0.936 (0.926–0.947)
SPISE high	0.886 (0.869–0.903)	0.935 (0.924–0.946)
	**Cut-off–sensitivity–specificity–Youden**	**Cut-off–sensitivity–specificity–Youden**
TyG Index high	-4.0-67.9-62.3-0.302	-1.0-71.0-70.2-0.412
METS-IR high	6.0-84.6-84.2-0.688	11.0-88.5-88.3-0.768
SPISE high	5.0-84.8-84.6-0.694	10.0-88.7-88.0-0.767

AUC: area under the curve. TyG: Triglyceride–Glucose Index. METS-IR: Metabolic Score for Insulin Resistance. SPISE: Single Point Insulin Sensitivity Estimator.

## Data Availability

The study data are stored in a database that complies with all security measures at ADEMA-Escuela Universitaria. The Data Protection Delegate is Ángel Arturo López González.
